# Endoscopic Hemostasis for Gastrointestinal Stromal Tumor Hemorrhage: A Case Series and Retrospective Analysis of Risk Factors

**DOI:** 10.1002/deo2.70158

**Published:** 2025-06-07

**Authors:** Karen Kimura, Chikamasa Ichita, Chihiro Sumida, Takashi Nishino, Miki Nagayama, Akiko Sasaki

**Affiliations:** ^1^ Gastroenterology Medicine Center Shonan Kamakura General Hospital Kanagawa Japan

**Keywords:** acute care surgery | endoscopic hemostasis | gastrointestinal hemorrhage | gastrointestinal stromal tumors | risk factors

## Abstract

**Objectives:**

Gastrointestinal stromal tumors (GISTs) are vascular tumors that can cause significant gastrointestinal hemorrhage. While endoscopic treatment is common for other hemorrhagic disorders, its role in GIST‐related hemorrhage remains unclear. This study presents a case series of five patients who underwent successful endoscopic hemostasis for upper gastrointestinal GISTs and explores potential risk factors for hemorrhage.

**Methods:**

This single‐center retrospective study included patients diagnosed with upper gastrointestinal GISTs between January 2013 and December 2022. Of the 61 eligible patients, the clinical courses of five patients who underwent endoscopic hemostasis were reviewed. In addition, an exploratory analysis was conducted to identify risk factors associated with GIST‐related hemorrhage by comparing hemorrhagic and non‐hemorrhagic groups using univariate analyses.

**Results:**

Five patients underwent successful endoscopic hemostasis using hypertonic saline‐epinephrine injection and/or clipping for hemorrhage from exposed vessels at the ulcer. Consequently, emergency surgery within 24 h was avoided in all cases, and elective surgery was conducted between 7 and 51 days. Additionally, ulcer formation (odds ratio [OR] 37.20; 95% confidence interval [CI] 2.40–582.00; *p* < 0.01), elevated white blood cell count (OR 1.05; 95% CI 1.01–1.09; *p* = 0.03), and elevated body mass index (OR 1.22, 95% CI 1.02–1.47, *p* = 0.03) were identified as risk factors for hemorrhage in the exploratory analysis.

**Conclusions:**

Endoscopic hemostasis may help avoid emergency surgery in GIST‐related hemorrhage when hemorrhage points are clearly visualized. Ulceration may serve as a predictive factor for hemorrhage; therefore, early surgical intervention should be considered in GISTs with ulceration.

## 1 Introduction

Gastrointestinal stromal tumors (GISTs) are highly vascularized tumors that can cause significant gastrointestinal hemorrhage, which is reported in approximately 23%–40% of all GIST cases [1, 2, 3, 4]. In some instances, severe hemorrhage necessitates emergency interventions such as transcatheter arterial embolization (TAE) or emergency surgery [5, 6, 7]. Although a meta‐analysis has summarized the relationship between gastrointestinal hemorrhage and prognosis in patients with GISTs [8], the included studies were largely conducted before 2020 and lacked data specific to Japanese patients. Furthermore, studies evaluating endoscopic hemostasis are extremely limited and outdated [9].

Additionally, while endoscopic treatment is widely used in other gastrointestinal hemorrhage settings, its effectiveness in GIST‐related hemorrhage remains unclear. However, in clinical practice, endoscopic hemostasis may serve as a temporary yet effective means to control hemorrhage and allow time for elective surgical management.

Therefore, in this study, we present a case series of five patients with upper gastrointestinal GISTs who underwent successful endoscopic hemostasis. We retrospectively examined the clinical course leading up to surgery and conducted an exploratory analysis of factors potentially associated with hemorrhage in patients with upper gastrointestinal GISTs.

## 2 Methods

### 2.1 Study Design and Patient

This retrospective case series and exploratory analysis were conducted at Shonan Kamakura General Hospital in Kanagawa, Japan. We reviewed the medical records of patients who underwent preoperative upper endoscopy and were finally diagnosed with upper gastrointestinal GIST between January 2013 and December 2022. Patients with hemorrhage after forceps biopsy or endoscopic ultrasound‐fine needle aspiration were excluded.

### 2.2 Characteristics of Endoscopic Hemostasis

We evaluated the outcomes and features of cases where primary endoscopic hemostasis was attempted for GIST hemorrhage. Details of these cases are presented as a case series.

### 2.3 Statistical Analysis

Exploratory analyses were performed to identify potential factors associated with hemorrhage in upper gastrointestinal GISTs. Continuous and categorical variables were compared using the Mann–Whitney U test and Fisher's exact test, respectively. Statistical significance was set at p < 0.05. Analyses were conducted using EZR version 1.55 [10], a graphical interface for R.

## 3 Results

### 3.1 Case Series of Endoscopic Hemostasis

Of the 70 patients diagnosed with GIST, 61 had tumors located in the upper gastrointestinal tract (esophagus, stomach, and duodenum). Among them, 18 patients presented with gastrointestinal hemorrhage, defined as melena, hematochezia, hematemesis, a ≥ 2 g/dL drop in hemoglobin, or the need for endoscopic hemostasis. Endoscopic examination was performed in 11 patients, of which five required and underwent endoscopic hemostasis.

#### 3.1.1 Case 1

**FIGURE 1:**
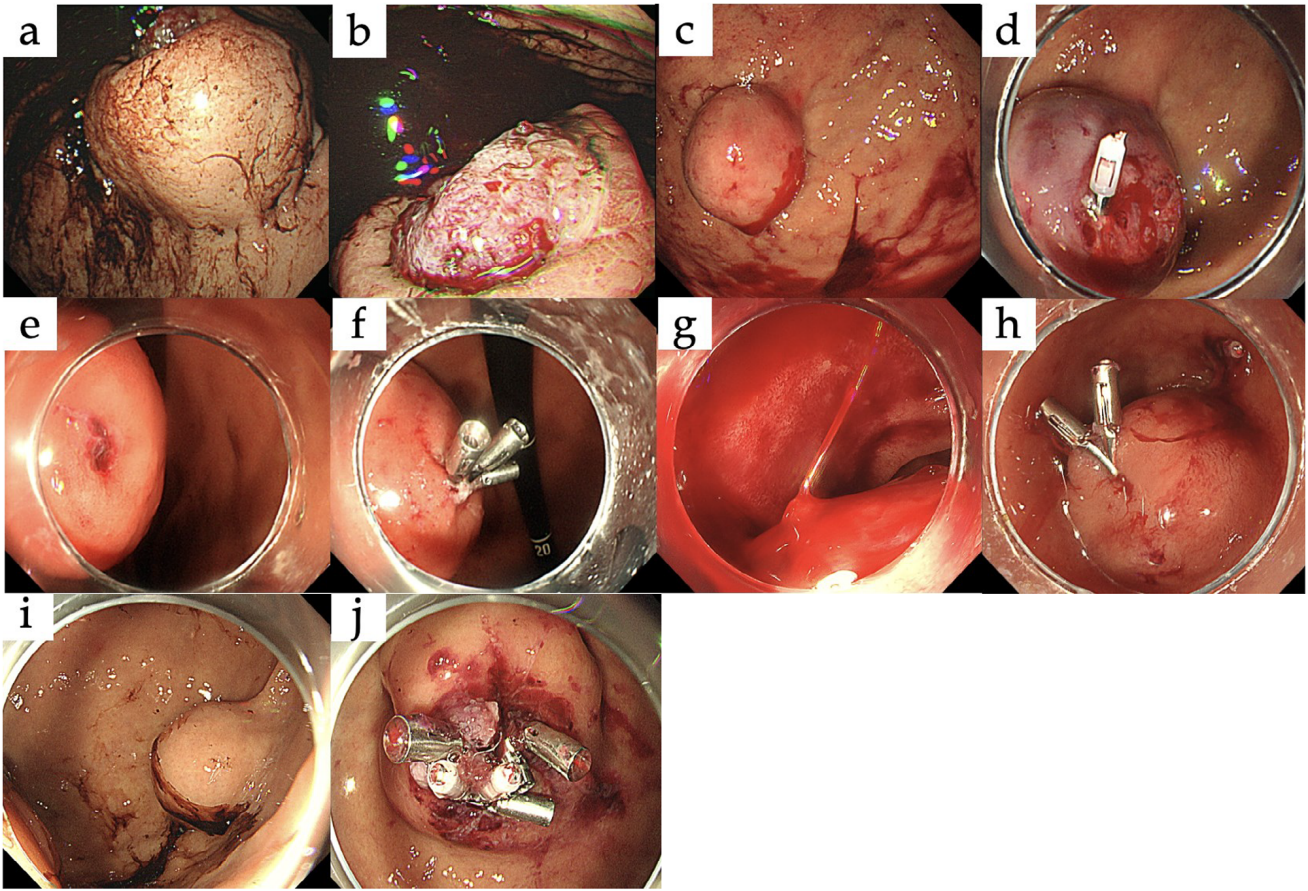
Representative cases of gastrointestinal stromal tumor (GIST) hemorrhage. (a, b) Lesion on the anterior wall of the gastric fundus. Oozing bleeding was observed from an exposed vessel at the ulcer, which was controlled with a hypertonic saline‐epinephrine (HSE) injection. (c, d) Lesion on the anterior wall of the gastric fundus. Oozing bleeding was observed from an exposed vessel at the ulcer, which was controlled with clipping. (e, f) Lesion on the greater curvature of the gastric body. Oozing bleeding was observed from an exposed vessel at the ulcer, which was controlled with clipping. (g, h) Lesion on the superior duodenal angle. Spurting bleeding was observed from an exposed vessel at the ulcer, which was controlled with clipping. (i, j) Lesion on the anterior wall of the gastric fundus. Oozing bleeding was observed from an exposed vessel at the ulcer, which was controlled with clipping.

A 44‐year‐old man, not taking antithrombotic medications, presented with melena. Laboratory tests showed a hemoglobin level of 11.3 g/dL. Upper gastrointestinal endoscopy revealed a 47 × 44 mm submucosal tumor with ulceration and a large ulcer located in the upper third of the stomach. An exposed vessel was observed at the ulcer, and the hemorrhage point was clearly visible, confirming the hemorrhage originating from the tumor (Figure 1a,b). Hemostasis was successfully achieved by hypertonic saline‐epinephrine (HSE) injection, with no further anemia progression. Laparoscopic partial gastrectomy was performed 32 days after endoscopic hemostasis and postoperative pathology confirmed the diagnosis of GIST.

#### 3.1.2 Case 2

A 71‐year‐old man undergoing aspirin therapy presented with hematemesis. Laboratory tests revealed anemia with a hemoglobin level of 6.9 g/dL. Upper gastrointestinal endoscopy revealed a 30 × 30 mm submucosal tumor with ulceration located in the upper third of the stomach. Although the ulcer was small and not clearly visible, an exposed vessel was identified, allowing successful hemostasis with HSE injection and clipping (Figure 1c,d). Despite these interventions, the anemia progressed, requiring transfusion, in addition to recurrent hematemesis, which indicated ongoing hemorrhage. Thus, open surgery was performed 3 days after the initial endoscopy. Postoperative pathology confirmed the diagnosis of GIST.

#### 3.1.3 Case 3

A 42‐year‐old woman, not taking antithrombotic medications, presented with hematemesis. Laboratory tests showed anemia with a hemoglobin level of 9.0 g/dL, but no signs of hypovolemic shock. Upper gastrointestinal endoscopy revealed a 38 × 34 mm submucosal tumor with ulceration located in the middle third of the stomach. The ulcer was small and the hemorrhage site was clearly visible showing active hemorrhage from an exposed vessel. Clipping was used to achieve hemostasis (Figure 1e,f) and laparoscopic pyloric‐sparing gastrectomy was performed after 7 days. Postoperative pathology confirmed the diagnosis of GIST.

#### 3.1.4 Case 4

A 75‐year‐old man on edoxaban presented with melena and hypovolemic shock, which responded to transfusion. Upper gastrointestinal endoscopy revealed a 25 × 20 mm submucosal tumor with ulceration located at the superior duodenal angle. Although the ulcer was large, the hemorrhage site was not clearly visible. Nonetheless, active spurting bleeding from an exposed vessel was successfully managed with endoscopic clipping (Figure 1 g,h). No recurrent hemorrhage occurred, and an open duodenal pyloromyotomy was performed 51 days after the endoscopy. Postoperative pathology confirmed GIST.

#### 3.1.5 Case 5

An 88‐year‐old woman undergoing aspirin therapy presented with hematemesis, but no signs of hypovolemic shock upon admission. Upper gastrointestinal endoscopy revealed a 25 × 17 mm submucosal tumor with ulceration located in the upper third of the stomach. The ulcer was large, the hemorrhage site was clearly visible. Oozing bleeding from an exposed vessel was successfully treated using endoscopic clipping (Figure 1i,j). No hemorrhage occurred, and laparoscopic partial gastrectomy was performed 11 days after endoscopy. Postoperative pathology confirmed GIST.

### 3.2 Clinical Outcomes and Endoscopic Findings

As summarized in Table 1, rehemorrhage occurred only in one patient with a small ulcer and unclear visualization of the hemorrhage point (Case 2). Whereas in the remaining patients, each having either a large ulcer or clear visualization of the exposed vessel, no rehemorrhage was observed until elective surgery.

**TABLE 1:** Characteristics of the five cases in which primary endoscopic hemostasis was achieved.

	Year	Age/Sex	Organ (Region)	Tumor diameter (mm)	Size of ulcer	Direct visualization of the vessel	Method of endoscopic hemostasis	Time to surgery	Surgical method
1	2013	44/M	Stomach (Upper)	47×44	Large	Clear	HSE Directorate injections	32 days	Laparoscopic partial gastrectomy
2	2017	71/M	Stomach (Upper)	30×30	Small	Unclear	HSE Directorate injections and clip hemostasis	Within 3 days	Open partial gastrectomy
3	2020	42/F	Stomach (Middle)	38×34	Small	Clear	Clip hemostasis	7 days	Laparoscopic pyloric‐sparing gastrectomy
4	2022	75/M	Duodenum (SDA)	25×20	Large	Unclear	Clip hemostasis	51 days	Open duodenal pyloromyotomy
5	2022	88/F	Stomach (Upper)	25×17	Large	Clear	Clip hemostasis	11 days	Laparoscopic partial gastrectomy

### 3.3 Supplementary Analysis: Risk Factor Evaluation

As a supplementary analysis, we explored the potential risk factors associated with hemorrhage in upper gastrointestinal GISTs. The results of the risk factor analyses are summarized in Table 2. Univariate analysis revealed an association between GIST and hemorrhage, ulceration (odds ratio [OR] 45.60, 95% confidence interval [CI] 5.16–402.00, *p* < 0.01), elevated white blood cells (WBC) (OR 1.06, 95% CI 1.03–1.10, *p* < 0.01), and elevated body mass index (BMI) (OR 1.22, 95% CI 1.02–1.47, *p* = 0.03).

**TABLE 2:** Risk factor analysis of gastrointestinal stromal tumor (GIST) hemorrhage.

	Univariate		
Characteristics	OR	95% CI	*p*‐Value
Age	0.97	0.92–1.03	0.32
Male	1.80	0.48–6.70	0.38
BMI	1.22	1.02–1.47	0.03
Underly conditions			
Coronary artery disease	1.27	0.25–6.52	0.77
Cerebral infarction	0	0–∞	0.99
Previous malignancy	1.73	0.18–17.00	0.64
Hypertension	1.65	0.33–8.29	0.54
Hyperlipidemia	1.55	0.35–6.97	0.56
Chronic kidney disease	1.07	0.06–18.6	0.96
Diabetes mellitus	1.69	0.24–12.00	0.60
Medications			
NSAIDs	3.23	0.45–23.20	0.24
Antithrombotic	2.98	0.67–13.30	0.15
Acid blockers	0.59	0.09–3.98	0.59
Tumor characteristics			
Location			
Esophagus and duodenum	1.00 (reference)	–	–
Upper third of the stomach	0.58	0.12–2.85	0.51
Middle third of the stomach	0.18	0.02–2.06	0.17
Lower third of the stomach	< 0.01	0‐∞	0.99
Diameter			
< 50, *n*, (%)	1.00 (reference)	–	–
≥ 50, *n*, (%)	0.88	0.20–3.76	0.86
Ulceration	45.60	5.16–402.00	<0.01
Blood sampling data			
White blood cell	1.06	1.03–1.10	<0.01
Platelet	1.04	0.96–1.13	0.29
Albumin	0.43	0.15–1.25	0.12
PT‐INR ≥ 1.5	0	0–∞	1.00
APTT (sec) ≥ 35	0	0–∞	1.00

Abbreviations: 95% CI, 95% confidence interval; GIST, gastrointestinal stromal tumor; IQR, interquartile range; NSAIDs, nonsteroidal anti‐inflammatory drugs.

## 4 Discussion

This case series shows that endoscopic hemostasis can avert emergent surgery in patients with GIST‐related hemorrhage. To the best of our knowledge, only Pih et al. have reported successful hemostasis in 32.6% of episodes with a rehemorrhage rate of 13.3% [9]. Similarly, in our five‐patient case series, hemostasis was achieved in all patients and none of them required surgery within 24 h. Additionally, rehemorrhage occurred only in the patient with a small ulcer and unclear endoscopic visualization of the vessel. These observations suggest that the combination of ulcer size and clarity of exposed‐vessel visualization may influence the risk of rehemorrhage after endoscopic hemostasis; however, future prospective studies are warranted to confirm this association. Although clipping was the most frequently used modality, its success depends on technical factors such as tumor location, ulcer morphology, fibrosis, and operator expertise. Thus, when these factors compromise clip placement, injection therapy (e.g., HSE) or thermal coagulation should be considered. Furthermore, if validated, the two endoscopic indicators identified—ulcer size and vessel visibility—could help clinicians decide when endoscopic hemostasis alone is sufficient and when more definitive or adjunctive measures are needed.

In addition, the univariate analysis identified ulcer formation, elevated WBC, and elevated BMI as risk factors for hemorrhage. Numerous case reports have identified hemorrhagic symptoms in GISTs [6, 11–15]; however, ulcer formation has not been previously identified as a risk factor for gastric GISTs [9]. For instance, Pih et al. and Wang et al. reported that a tumor size >5 cm was the main predictor of hemorrhage in GISTs [9, 16]. In contrast, our findings suggest that ulcer formation plays a more significant role in predicting hemorrhage in GISTs compared to tumor size (OR 9.84, 95% CI 2.07–46.70). This difference may be attributed to differences in study populations and diagnostic criteria. The underlying mechanism can be explained as follows: the destruction of the mucosal barrier through ulcer formation exposes the underlying blood vessels to gastric acid stimulation, causing the vessels to become exposed and rupture, resulting in hemorrhage [17]. Thus, the tumor size was not identified as a risk factor in our study, because as the tumor diameter increases, tumor invasion into the mucosa and mucosal damage become more probable, thereby promoting ulcer formation and potentially becoming a critical factor for hemorrhage [9, 12, 18]. However, data identifying ulcer formation as the primary determinant of hemorrhage remains limited. Consequently, in this regard, our results are relevant as they emphasize the significance of ulcer formation as a key risk factor, suggesting that it plays a major role in the occurrence of hemorrhage. Therefore, in cases of GIST with ulceration, early surgical intervention should be considered before hemorrhage occurs. If hemorrhage does occur, attempting endoscopic hemostasis may help avoid emergency surgery. Although the exact mechanism of how elevated WBCs impact hemorrhage is unclear, it may reflect a systemic inflammatory response to tumor‐related injury or represent a secondary reaction to acute hemorrhage. Similarly, while elevated BMI was also identified as a risk factor in the univariate analysis, this finding should be interpreted with caution due to the limited sample size and lack of supporting evidence in previous studies.

Nonetheless, this study has some limitations that require consideration. First, the retrospective design and single‐center setting may have restricted the generalizability of the findings. Second, the small sample size and single‐center design limit the generalizability of the findings. As hemorrhage was often diagnosed during endoscopy, the univariate analysis does not represent a true predictive model. These results should be interpreted cautiously, and validation through multicenter studies with larger cohorts is needed. Furthermore, to the best of our knowledge, no study has specifically examined endoscopic hemostasis or lesion characteristics in GIST‐related hemorrhage, therefore, we believe our findings offer valuable insights that may contribute to future research in this area.

In conclusion, endoscopic hemostasis may be an effective strategy to prevent emergency surgery in patients with GIST‐related hemorrhage, particularly when ulcer formation, exposed vessels, and active hemorrhage are identified and the hemorrhage point is clearly visualized. In addition, ulceration may be a risk factor for hemorrhage, therefore, in cases of GIST with ulceration, early surgical intervention should be considered before hemorrhage occurs.

## Conflicts of Interest

The authors declare no conflicts of interest.

## Ethics Statement

This study was approved by the Institutional Review Board of Mirai Iryo Research Center Inc., Shonan Kamakura General Hospital (institutional ID: CRB3210004; TGE02141‐024), and was performed in accordance with the principles of the Declaration of Helsinki.

## Consent

Informed consent was presumed unless patients opted out of the study.

## Clinical Trial Registration

N/A.
